# HHV-8-unrelated primary effusion-like lymphoma associated with clonal loss of inherited chromosomally-integrated human herpesvirus-6A from the telomere of chromosome 19q

**DOI:** 10.1038/srep22730

**Published:** 2016-03-07

**Authors:** Enjie Zhang, Victoria E. Cotton, Alberto Hidalgo-Bravo, Yan Huang, Adam J. Bell, Ruth F. Jarrett, Gavin S. Wilkie, Andrew J. Davison, Ellie P. Nacheva, Reiner Siebert, Aneela Majid, Inga Kelpanides, Sandrine Jayne, Martin J. Dyer, Nicola J. Royle

**Affiliations:** 1 Department of Genetics, University of Leicester, Leicester, LE1 7RH, UK; 2 MRC – University of Glasgow Centre for Virus Research, Glasgow G61 1QH, UK; 3 Cytogenetics Laboratory, Royal Free London NHS Foundation Trust, London, NW3 2PF, UK; 4 Institute of Human Genetics, Christian-Albrechts-University Kiel & University Hospital Schleswig-Holstein, Campus Kiel, Schwanenweg 24, D-24105 Kiel, Germany; 5 Ernest and Helen Scott Haematological Research Institute, Department of Cancer Studies, University of Leicester, Leicester, LE1 7RH, UK

## Abstract

Primary effusion lymphomas (PEL) are associated with human herpesvirus-8 (HHV-8) and usually occur in immunocompromised individuals. However, there are numerous reports of HHV-8-unrelated PEL-like lymphomas with unknown aetiology. Here we characterize an HHV-8-unrelated PEL-like lymphoma in an elderly woman who was negative for human immunodeficiency viruses 1 and 2, and hepatitis B and C. The woman was, however, a carrier of an inherited-chromosomally-integrated human herpesvirus-6A (iciHHV-6A) genome in one 19q telomere. The iciHHV-6A genome was complete in blood DNA, encoding a full set of protein-coding genes. Interestingly, the entire iciHHV-6A genome was absent from the HHV-8-unrelated-PEL-like lymphoma cells despite retention of both copies of chromosome 19. The somatic loss of the 19q-iciHHV-6A genome occurred very early during lymphoma development and we propose it occurred via telomere-loop formation and excision to release a circular viral genome that was subsequently lost. Whether release of the HHV-6A genome from the telomere contributed to lymphomagenesis, or was coincidental, remains unclear but this event may have deregulated the expression of HHV-6A or 19q genes or else disrupted telomere function. To establish the frequency and importance of iciHHV-6 loss from telomeres, the HHV-6 copy number should be assessed in tumours that arise in iciHHV-6 carriers.

Primary effusion lymphoma (PEL) is a B cell malignancy, usually arising in immunocompromised, human immunodeficiency virus (HIV)-infected individuals, associated with latent infection of human herpesvirus-8 (HHV-8; also known as Kaposi’s sarcoma-associated herpesvirus)^1^,^2^. PEL is confined anatomically to the serous cavities, with no lymph nodal involvement, and is a plasmablastic disease associated with a very poor prognosis. Initially, PEL was thought to be universally associated with HHV-8 infection. However, it has become clear more recently that primary effusion-like lymphoma may also occur in HIV-negative individuals who are not overtly immunosuppressed, and in the absence of HHV-8 infection^3^. These lymphomas have collectively been termed HHV-8-unrelated PEL-like lymphomas^4^.

HHV-8-unrelated PEL-like lymphomas are heterogeneous but they differ immunophenotypically and genotypically from HHV-8-positive PEL and may more closely resemble diffuse, large B-cell lymphoma (DLBCL) or Burkitt lymphoma (BL), or even plasmacytoid malignancies and may constitute a rare but distinct pathological entity (reviewed in^5^,^6^). The mature B-cell phenotype of most cases of HHV-8-unrelated PEL-like lymphoma with expression of CD19 and CD20 is strikingly different from regular HHV-8-associated PEL, which lack expression of B-cell antigens. For reasons that are unclear, most reported cases have involved elderly or very elderly patients from Japan. Patients who are able to tolerate systemic chemotherapy appear to do well, again in contrast to patients with regular PEL.

HHV-6A and HHV-6B are closely related viruses but distinct species of the subfamily *Betaherpesvirinae*, family *Herpesviridae*^7^. In western societies, infection typically occurs in early childhood in over 95% of individuals. HHV-6B usually causes a mild, febrile illness (roseola infantum or exanthema subitum) in children, whereas HHV-6A has not been associated with particular symptoms. HHV-6A or -6B can persist in a latent form in most individuals but, if reactivation occurs, it can have severe consequences, particularly in immunocompromised individuals^8^. The HHV-6A and -6B genomes contain over 100 open reading frames (ORFs) encoding 97 genes that are expressed in an ordered manner during active infection^9^,^10^,^11^. The roles of many HHV-6 genes are poorly understood but like other herpesviruses, HHV-6 is able to circumvent the pathway that results in p53 mediated cell cycle arrest and so is able to replicate^12^. Several HHV-6 genes, including DR6/DR7 (ORF-1), U14 and U19, encode proteins that interact with p53 directly of indirectly^13^,^14^,^15^,^16^. Interestingly the U14 protein has been detected with p53 in virions and its role is under intense investigation^14^,^17^,^18^. Although *in vitro* experiments suggest that HHV-6 DNA can transform cells in culture^19^, investigation of the relationship between HHV-6 and cancer is controversial as a clear pathogenic role has not been demonstrated 20^,^^21^. HHV-6 DNA, viral proteins or particles are often detected in haematological and other malignancies, which at least demonstrates that HHV-6 is an opportunistic pathogen that can readily reactivate in some cancer settings^20^.

A distinctive feature of both viruses is the capacity for integration into telomeres, which consist of repetitive (TTAGGG)_n_ sequences that form nucleoprotein-capping structures at the ends of chromosomes^22^. The rate of germline telomeric integration is unknown, but a small proportion of individuals, approximately 0.8% of the London-based population in the UK, have inherited a copy of chromosomally-integrated HHV-6 (ciHHV-6)^23^, and can transmit it to their children. Integration is mediated by HHV-6 sequences (T1 and T2), which are homologous to telomeric DNA and located within both copies of the terminal direct repeats (DR)^24^,^25^,^26^. The two DRs are termed DR_L_ and DR_R_, and are located at the left and right ends of the annotated viral genome, respectively.

Recently, it has been shown that inherited ciHHV-6 (iciHHV-6) represents a state of latency, as full viral reactivation was detected in an immunocompromised child with iciHHV-6A and hemophagocytic syndrome^27^. In addition reactivated virus has been shown to pass from iciHHV-6 mothers to non-iciHHV-6 children via the placenta^28^. This raises the possibility that telomeric integration is also a form of latency for the majority of the population, with lifelong HHV-6 latency occurring in a small proportion of somatic cells. Here, we describe a woman who was diagnosed with PEL-like lymphoma in the absence of infection by HHV-8, HIV and other viruses, and who had iciHHV-6A in one 19q telomere. We show that the HHV-6A genome was absent from the HHV-8-unrelated PEL-like lymphoma cells, and propose that telomere-loop (t-loop) formation within the iciHHV-6 genome followed by excision may have released the viral genome from the telomere^24^,^25^,^29^,^30^. Loss of the iciHHV-6 genome could either have been a coincidental ‘passenger’ event during cancer initiation or alternatively the disruption caused by loss of the viral genome from the telomere may have contributed to the development of the HHV-8-unrelated PEL-like lymphoma by a novel mechanism.

## Results

### Patient information, diagnosis and treatment

A previously well, 73 year-old lady (numbered 1500), with no prior history of infections, presented with an 8-week history of left-sided pleuritic chest pain and breathlessness. CT scan revealed a medium-sized pleural effusion with no lymphadenopathy or extranodal masses within the thorax, abdomen or pelvis. Drainage of the pleural fluid showed pleomorphic lymphoid blasts (CD45+, CD20+, CD79a+, CD3−, CD10−). Bone marrow aspirate and trephine were normal and additional clinical details are given in the materials and methods. Viral serological studies showed that a peripheral blood sample was negative for HIV-1 and HIV-2, hepatitis B virus and hepatitis C virus. PCR assays for HHV-8 and EBV did not detect either viral genome in DNA extracted from a peripheral blood sample (taken at diagnosis) or in the pleural fluid sample (predominantly lymphoma cells). Consequently a diagnosis of HHV-8 unrelated PEL-like lymphoma was made.

Chromosome analysis of R-banded metaphases from short-term cultures of the pleural fluid revealed the karyotype: 48~49,XX, +X,der(2)t(2;12)(q14;q12),der(3)t(2;3)(q13;q11),t(3;22)(q27;q11),del(4)(q26q28),t(6;12)(p23;p12),del(6)(q14q22),add(7)(p14), +11,del(11)(q14), +12,der(12)t(4;12)(q13;q23~23)del(4)(q24q28),i(21)(q10)[cp30] (Supplementary Fig. S1). The t(3;22)(q27;q11), was confirmed by fluorescent *in situ* hybridization (FISH) analyses using a *BCL6* break-apart and spanning probe^31^ as well as break-apart and spanning assays for the *IGL* locus^32^. The pleural effusion completely resolved following six cycles of R-CHOP therapy, and the patient remained in continuous, complete remission for the next nine years, eventually dying from complications of late-onset diabetes with no evidence of recurrent lymphoma.

### Identification of iciHHV-6 in the patient and siblings

Seeking a potential viral aetiology for the HHV-8-unrelated PEL-like lymphoma, further viral PCR studies were performed and the HHV-6 U11 gene was detected at approximately 10^6^ viral copies/μg of DNA in peripheral blood, consistent with one copy of the viral genome/cell. DNA sequence analysis showed that HHV-6A was present, rather than HHV-6B.

**Figure 1:**
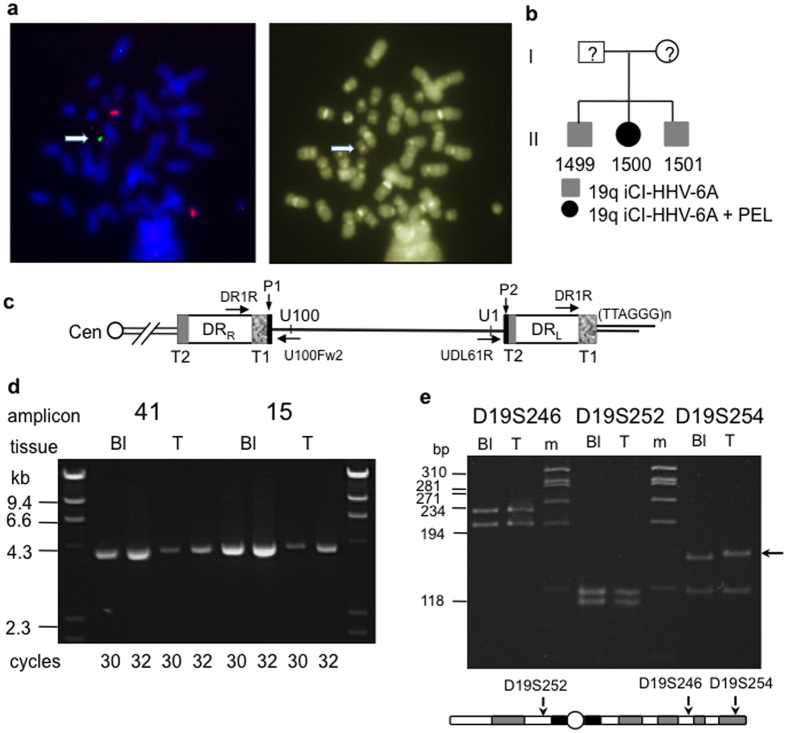
iciHHV-6A at 19q in three siblings and loss from the HHV-8-unrelated PEL-like lymphoma. (**a**) FISH on metaphase chromosomes from 1500-Bl (blood). The green labeled HHV-6 probe showed HHV-6 sequence at the telomere of chromosome 19q, a probe for centromere 20 (red signals) served as a control (**b**). Pedigree of family with iciHHV-6A at 19q. (**c**) Organisation of iciHHV-6A in the 19q telomere. Diagram shows location of priming sites for DR1R, U100Fw2 and UDL61R. T1 and T2 are the imperfect and perfect arrays of viral encoded (TTAGGG)_n_ repeats respectively. P1 and P2 show the locations of the PAC1 and PAC2 sequences retained in the integrated viral genome. (**d**) Examples of semi-quantitative analysis of two HHV-6A amplicons (41 and 15) in 1500-Bl and 1500-T (pleural fluid containing HHV-8-unrelated PEL-like lymphoma cells). (**e**) 1500 was heterozygous at three STRs on chromosome 19 (D19S252, D19S246, D19A254) in blood DNA (Bl) and heterozygosity is retained in the lymphoma (T). Black arrow shows the mutated D19S254 allele.

The presence of HHV-6A at one copy per cell suggested iciHHV-6 rather than viral reactivation. This was confirmed by metaphase FISH, which showed the iciHHV-6 to be present at a telomere of chromosome 19q (Fig. 1a). HHV-6 sequences were also detected in hair follicle DNA from the patient. The patient’s two elderly brothers (1499 and 1501) were also positive for iciHHV6A at 19q (Fig. 1b); both were well and lacked serious medical problems. Telomeric integration was confirmed in all three siblings by using STELA with the DR1R primer^25^. The generation of STELA products of variable length in each donor demonstrated the presence of a telomere on the end of DR_L_ in the ciHHV-6A genome (Fig. 1c).

### Loss of ciHHV-6 in the primary tumour

FISH using HHV-6 probes failed to detect viral integration in the HHV-8-unrelated PEL-like lymphoma cells in the pleural fluid (1500-T). In addition, semi-quantitative PCR analysis of amplicons covering the full length of the HHV-6A genome showed reduction of viral sequences in pleural fluid (1500-T) compared with blood DNA (1500-Bl; Fig. 1d and Supplementary Fig. S2). We verified that the blood and tumour sample were from the same individual (1500) using genetic profiling with the hypervariable minisatellites, MS1 (D1S7) and B6.7^33^,^34^ (Supplementary Fig. S1). The loss of HHV-6 sequence from 1500-T was confirmed by ddPCR analysis of HHV-6A targets relative to the human RPP30 gene. Thus, copy number analysis of the HHV-6A U38 and U7 sequences showed that the HHV-6A genome was present at 0.1 copies/cell in the pleural fluid sample in comparison to 1.1 copies/cell in blood DNA (1500-Bl) and similar levels in the two brothers, 1499-Bl and 1501-Bl (Table 1). The expected 2:1 ratio of DR6:U38 sequences in the HHV-6A genome was constant in the 1500-Bl and 1500-T samples. This suggests that the entire HHV-6A genome with both DR regions was lost from the lymphoma, as there is no evidence that one DR region was retained in the telomere^25^. The 1500-Bl and 1500-T were heterozygous at three polymorphic STRs on chromosome 19, indicating that loss of the iciHHV-6 genome from the HHV-8-unrelated PEL-like lymphoma had not occurred simply through loss of the chromosome carrying the integration (Fig. 1e). One allele at the distal STR, D19S254 (1.4 Mb from the 19q telomere), had undergone a somatic mutation in the lymphoma that also appeared clonal in origin.

### The 19q iciHHV-6A genome contains a full set of protein-coding regions

The iciHHV-6A genome was oriented with DR_L_ located towards the terminus of the chromosome, as expected (Fig. 1c)^24^,^25^. The DR_L_-T2 region was 84 bp in size, comprising 14 (TTAGGG) repeats in 1499-Bl, 1500-Bl and 1501-Bl, and was located adjacent to the P2 packaging sequence in DR_L_. The terminal DR_L_-P1 packaging sequence was absent and replaced by (TTAGGG)_n_ repeats, whereas the internal DR_R_-P1 packaging sequence was retained and located adjacent to a 2.1 kb DR_R_-T1 region (Fig. 1c). In summary, the terminal P1 and P2 sequences of DR_L_ and DR_R_ respectively were lost from the ends of the HHV-6A genome upon integration into the 19q telomere, as shown previously^24^,^25^.

Overlapping amplicons covering the iciHHV-6A genome were generated from 1500-Bl, the residual viral DNA in 1500-T, and 1501-Bl. All the amplicons were the expected sizes, indicating the absence of large insertions or deletions disrupting the viral genome (Supplementary Fig. S3). The amplicons were pooled from each sample, and the sequence of the ciHHV6-A genome was determined by using next-generation sequencing methods. Three regions (T1 and other repeat regions) could not be formally closed because of their lengths. Assembly of sequence data for 1500-Bl and 1500-T showed that the ciHHV-6 genomes in these samples were identical to those of 1501-Bl. The sequence was deposited in GenBank under accession number KT355575.

Comparisons with the annotation of HHV-6A strain U1102 (NC_001664) indicated that the sequence contained the full set of protein-coding regions, although gene U86 was not formally validated because of the presence of a large repeat array in this protein-coding region. Overall, the integrated genome was more distantly related (97.2–97.3% identity) to HHV-6A strains U1102 (NC_001664), GS (KC465951 and KJ123690) and AJ (KP257584) than these are from each other (98.4–99.1%). The sequence across the start of gene U83 is the same as in strain U1102, indicating that the iciHHV-6A in this family encodes both the long and short forms of the U83 chemokine^35^. The U14 protein, which interacts with p53^14^,^17^,^18^ is almost identical in amino acid sequence in strains U1102 and GS (99.3% identity), but interestingly more diverged in 1500 (93.4% identity to strains U1102 and GS). Similarly, gene U90, which is located in the immediate early 1 locus and plays important roles in active infection and regulation of latency^36^, shows a greater amino acid sequence divergence between 1500 and strain U1102 (91.6% identity) or GS (92.3% identity) than between strains U1102 and GS (95.3% identity). Further investigation will be required to determine whether the sequence divergence of these or other HHV-6A proteins influences the probability of viral genome release, reactivation or contribution to oncogenesis.

### Telomere length analysis and viral excision

**Figure 2:**
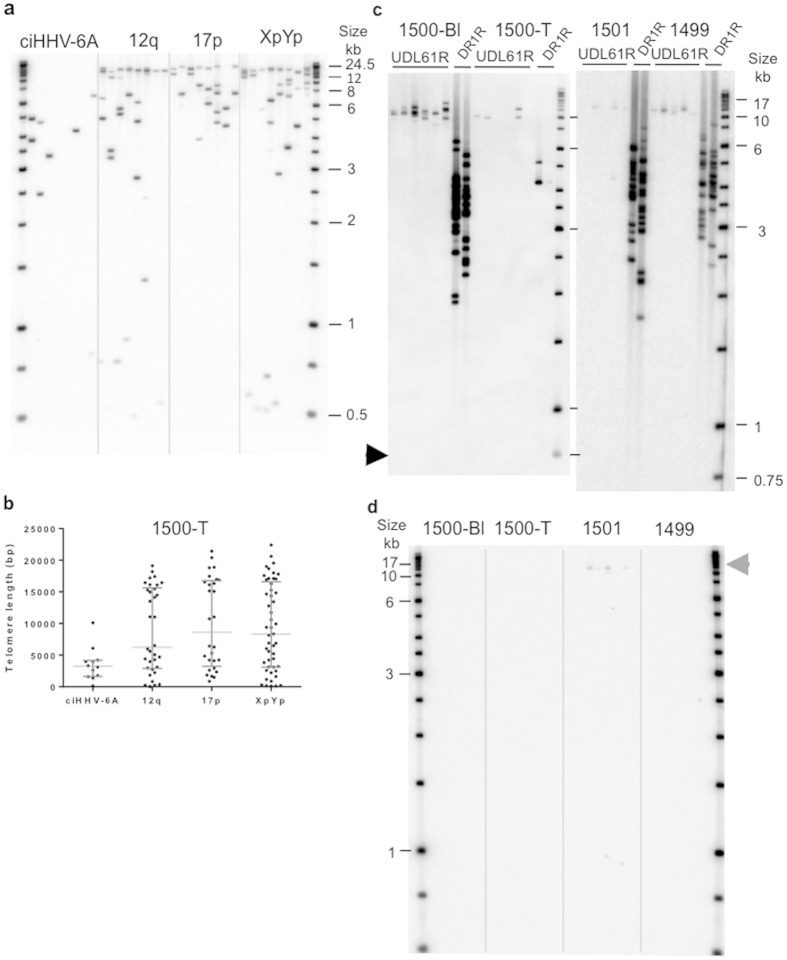
Telomere and iciHHV-6A analysis in the lymphoma and blood DNAs. (**a**) An example STELA blot showing amplified telomere molecules at iciHHV-6A (DR1R primer), 12q, 17p and XpYp in the pleural fluid (1500-T). (**b**) Scatter plots of the compiled 1500-T telomere length data showing median length and inter-quartile ranges. (**c**) PCR-based truncation assay using the UDL61R primer in STELA in the patient’s blood (1500-Bl) or pleural fluid (1500-T) and in DNA from the two brothers (1499 and 1501). The arrow shows the expected location of DR_L_-T2 truncation products (**d**) Amplification with U100Fw2 and UDL61R primers generated products (grey arrow head) in the DNA from brother 1501. These amplicons show the presence of low abundance extra-chromosomal circular HHV-6A molecules.

Using STELA with the DR1R primer, we have shown previously that the 19q iciHHV-6A-associated telomere was the shortest measured in blood DNA from 1499, 1500 and 1501, when compared to telomeres at 12q, 17p and XpYp^25^. Here, we measured the length of these telomeres in the pleural fluid sample, 1500-T (Fig. 2a, b). The 12q, 17p and XpYp telomeres were all significantly longer in 1500-T than in 1500-Bl (Table 2), indicating lengthening in the HHV-8-unrelated PEL-like lymphoma cells. Limited data were obtained from the iciHHV-6A-associated telomere in the 1500-T DNA, consistent with the low copy number of the HHV-6A genome in the cells from the pleural fluid. In contrast to the 12q, 17p and XpYp telomeres, the iciHHV-6A-associated telomere in the pleural fluid (1500-T) was not lengthened (median length 3.1 kb; Table 2) but had the same length to that measured in blood DNA, 1500-Bl (p = 0.8694). This is consistent with the iciHHV-6A-associated telomere molecules in the pleural fluid originating from normal cells in this sample rather than the HHV-8-unrelated PEL-like lymphoma cells.

### Investigation of iciHHV-6A stability in the family

We have shown previously that iciHHV-6B can be unstable and undergo truncation events, releasing partial or complete circular molecules of the viral genome^25^. We proposed that such excision events are mediated by t-loop formation between the single-stranded telomeric overhang and internal telomere-like repeats at T2 and T1 in DR_L_ or DR_R_. Here, we did not detect ciHHV-6A truncation events at DR_L_-T2 in 1500-Bl, 1500-T, 1499-Bl or 1501-Bl (Fig. 2c). However, PCR using primers UDL61R and U100Fw2, which, because of their locations and orientations, do not amplify from the iciHHV-6A, detected large, low abundance molecules in 1501-Bl (Fig. 2d). These amplicons included a single DR containing both packaging sequences (P1 and P2), and are likely to have arisen via recombination between DR_L_ and DR_R_ in the ciHHV-6A genome, resulting in release of the viral genome as a circular molecule.

## Discussion

PEL represents a distinctive form of B-cell malignancy. It arises in the context of HHV-8 infection, mostly in HIV-infected individuals and the tumour cells contain HHV-8 genomes with frequent EBV co-infection. In contrast, HHV-8-unrelated PEL-like lymphoma can arise in normal or in immunocompromised patients and they have a different immunophenotype from HHV-8-associated PEL^5^,^6^. The aetiology of HHV-8-unrelated PEL-like lymphomas is unknown. Here, we have reported an HHV-8-unrelated PEL-like lymphoma, arising in a non-immunocompromised although elderly woman, which is associated with iciHHV-6A at a 19q telomere. The iciHHV-6A in the patient, 1500, and her brothers was intact, although, as expected, it lacked the terminal DR_L_-P1 and DR_R_-P2 packaging and cleavage sequences (Fig. 1c)^24^,^25^. Sequence analysis showed that the ciHHV-6A genomes were identical in the three siblings and comprised a full set of ORFs when compared to the reference HHV-6A strain U1102. The iciHHV-6A genome in this family is slightly more diverged from the HHV-6A strains sequenced previously (U1102, GS and AJ^37^,^38^) than they are from each other. Further investigation is needed to determine whether this is functionally significant.

Using semi-quantitative PCR and ddPCR, we showed that the entire viral genome is reduced from approximately 1 to 0.1 copies/cell in the HHV-8-unrelated PEL-like lymphoma sample (1500-T). Moreover, the residual viral DNA is associated with a short telomere, as detected in 1500-Bl DNA^25^, whereas three other telomeres (12q, 17p and XpYp) were lengthened in 1500-T. Telomere lengthening and maintenance is a hallmark of cancer cells as it is required for replicative immortality. The majority of cancers (approximately 85%) express telomerase, which stabilizes and maintains telomeres within in a fairly discrete length range. In contrast, approximately 15% of cancers activate the Alternative Lengthening of Telomeres (ALT) mechanism. Telomeres in ALT^+^ cells are very heterogeneous in length and can show large fluctuations in length, as a consequence of the recombination-based mechanism that involves strand invasion and copying between telomeres^39^. From the telomere length profiles we observed in the HHV-8-unrelated PEL-like lymphoma sample (1500-T), we infer that telomerase had been activated, as seen in most hematological malignancies.

Together the low copy number of the iciHHV-6A genome and the absence of lengthening at the associated telomere, suggests that the residual ciHHV-6A DNA in the pleural effusion sample (1500-T) comes from normal cells. Loss of the iciHHV-6A genome in the HHV-8-unrelated PEL-like lymphoma was not a result of loss of heterozygosity, as both copies of chromosome 19 were retained. Altogether, the data indicate that loss of the 19q iciHHV-6A occurred very early during HHV-8-unrelated PEL-like lymphoma development.

The presence of iciHHV-6 causes instability in the telomere carrying the insertion, and this can result in truncations at DR_L_-T2. We have proposed that these truncations may occur via t-loop formation at DR_L_-T2, accompanied by excision of the aberrant t-loop^25^. We did not detect DR_L_-T2 truncations in 1500 or her brothers (Fig. 2c), but T2 in this 19q-iciHHV-6A is short, just (TTAGGG)_13_, which may reduce the frequency of t-loop formation in this region. Further work is ongoing to understand the relationship between the length of T2 and frequency of DR_L_-T2 truncations. We assume similar truncation events occur at the internally located DR_R_-T1 (Fig. 1c) but we cannot use PCR-based assays to distinguish truncations at DR_R_-T1 from STELA products that represent the telomere at the end of DR_L_-T1, because the DR regions are identical. The U100Fw2-UDL61R PCR assay identified amplicons derived from recombination between DR_L_ and DR_R_ in 1501 but not the other two siblings (Fig. 2d), indicating that the proposed viral genome excision events can occur at a low frequency in blood DNA.

As the heterozygosity and cytogenetic analyses (Supplementary Fig. S1) show that the two copies of chromosome 19 are intact in the HHV-8-unrelated PEL-like lymphoma (1500-T), we propose that loss of the iciHHV-6A genome was not a result of generalised genome instability during lymphomagenesis. Rather, loss of the iciHHV-6A appears to have been a specific, clonal event that occurred very early during the lymphoma development. This event could have been mediated by t-loop formation between DR_L_ and DR_R_ leading to excision^29^,^30^ and release of the viral genome from the telomere in a somatic cell of patient 1500. It has been shown that iciHHV-6 can reactivate, leading to expression of viral genes and production of viral particles^27^,^28^, but it is unclear whether the 19q-iciHHV-6A or the release of the viral genome from the telomere contributed directly to lymphoma formation in this woman. On epidemiological grounds, given the frequency of its occurrence in the general population, it seems unlikely that iciHHV-6 *per se* plays a significant oncogenic role. Furthermore, recent surveys indicate that iciHHV-6 carriers do not have an increased risk of developing classical Hodgkin Lymphoma^40^ nor childhood acute lymphoblastic or myeloid leukemias^41^,^42^. However, somatic loss of the 19q-iciHHV-6A in the patient described here could have resulted in the transient presence of a circular HHV-6A genome and expression of viral genes that contributed to disruption of the cell cycle in this elderly patient. Alternatively and more likely, loss of the iciHHV-6A genome could have disrupted the telomere function or the expression of adjacent 19q subtelomeric genes^43^ and so contributed to the initiation of the HHV-8-unrelated PEL-like lymphoma, without HHV-6A gene expression or viral reactivation. By extrapolation, excision of ciHHV-6 by telomere-driven processes from somatic cells in other iciHHV-6 carriers, or perhaps from somatic cells harbouring latent ciHHV-6 in non-iciHHV-6 individuals, could have broader effects that may depend on the chromosome carrying the integration, the telomere length in the tissue where somatic loss occurs and therefore the age of the individual. To determine whether somatic loss of iciHHV-6 is a novel mechanism that can contribute to oncogenesis, we suggest that copy number of HHV-6 should be assessed, using sensitive ddPCR, in tumours arising in iciHHV-6 carriers.

## Materials and Methods

### Patient information and additional clinical details

The patient, 1500, presented with left-sided pleuritic chest pain and breathlessness. Initial investigations revealed the patient’s haemoglobin at 12.5 g/L, a white cell count of 4.2 × 10^9^/L (neutrophils 3.28 × 10^9^/L, lymphocytes 0.7 × 10^9^/L), platelet count of 226 × 10^9^/L, and normal lactic dehydrogenase, kidney and liver function tests. Peripheral blood immunophenotyping at diagnosis showed a moderate lymphopenia with mild CD4+ T-cell lymphopenia (a CD4 count of 0.32 × 10^9^/L; normal range 0.5–1.8 × 10^9^/L). The CD4:CD8 ratio was normal, and peripheral blood B cells were polyclonal. Serum IgG levels were slightly reduced (4.4 g/L; normal range 6–16 g/L), but IgM and IgA levels were normal. A low level of IgG kappa paraprotein (0.4 g/L) was also detected.

In line with the presence of t(3;22)(q27;q11) in the karyotype obtained from short-term cultures of the pleural fluid, fluorescent *in situ* hybridization (FISH) analyses using a *BCL6* break-apart (Abbott Laboratories) and spanning probe^31^ as well as break-apart and spanning assays for the *IGL* locus^32^, confirmed chromosomal breakpoints involving *BCL6* and *IGL* respectively as well as *BCL6-IGL* fusion in the vast majority of cells. There was no evidence for breakpoints or amplifications involving *MYC* on chromosome 8q24 or the *IGH* locus on 14q32, using break-apart probes for both loci (Abbott Laboratories).

Peripheral blood (1500-Bl) and PEL (1500-T) samples from the patient 1500 and DNA samples from her two brothers (1499 and 1501) were obtained for further analysis following informed consent and approval from the local Research Ethics Committee (Protocol 06/Q2501/122) in accordance with approved guidelines (Declaration of Helsinki).

### Detection of HHV-6 by PCR and cytogenetic analysis

HHV-6 DNA was detected, by quantitative PCR of the U11 gene, in a peripheral blood sample from 1500 at diagnosis (Micropathology Ltd, Coventry, UK), and showed approximately 10^6^ viral copies/μg of DNA, consistent with one copy of the viral genome/cell. DNA sequence analysis showed that HHV-6A was involved, rather than HHV-6B. HHV-6 sequences were detected in metaphase chromosomes by FISH^44^,^45^ using pooled clones: pMF311–12, pMF210–8 and pMF220–21^46^ prepared and labeled by standard procedures^32^.

### DNA sequencing of iciHHV-6 using next-generation technologies

The iciHHV-6A genome in each DNA sample was amplified using 32 pairs of overlapping primers (Sup. Table 1). Each 10 μl PCR reaction contained 7.5 ng genomic DNA, 0.9 μl PCR buffer [final concentration: 1 mM each dNTP, 45 mM Tris-HCl (pH 8.8), 11 mM (NH_4_)_2_SO_4_, 113 μg/ml bovine serum albumin, 4.5 mM MgCl_2_, 6.7 mM 2-mercaptoethanol and 4.4 μM EDTA (pH 8.0)]^47^,^48^, 0.3 μM each primer (forward and reverse), 0.1 μM Tris and 0.1 μl of an 8:1 ratio of *Taq* polymerase (5 U/μl, Kapa Biosystems) and *Pwo* polymerase (2.5 U/μl, Genaxxon Bioscience). Thermal cycling conditions were 96 °C for 2 minutes, followed by 35 cycles (32 cycles for DR) of 96 °C for 10 s, 52–62 °C for 30 s and 68 °C for 4–10 minutes, and then a final extension step at 68 °C for 10 minutes.

The 32 amplicons from each DNA sample were pooled and used to prepare barcoded libraries and templates according to the manufacturer’s protocols for sequencing on a MiSeq sequencer (Illumina) or an IonTorrent personal genome sequencer (Life Technologies). The MiSeq read data for 1501-Bl were assembled *de novo* into contigs by using ABySS v. 1.5.2^49^. A draft sequence was generated using scaffold builder^50^ to order the contigs against the reference HHV-6A strain U1102 sequence (NC_001664). Gaps were closed by using Megamerger^51^, GapFiller v. 1–11^52^ and custom Perl scripts, or by manual Sanger sequencing. The integrity of the resulting sequence was verified by aligning it against the read data by using BWA v. 0.6.2-r126^53^ and visualizing the alignment by using Tablet v. 1.13.08.05^54^. Data for 1500-Bl and 1500-T were generated on both the MiSeq and IonTorrent platforms, and were aligned against the 1501-Bl data by using BWA and visualized by using Tablet.

### Semi-quantitative analysis of HHV-6A amplicons

The DNA concentration was determined from multiple spectrophotometer readings (OD_260_; NanoDrop 1000, Thermo Scientific). The 10 μl PCRs contained 10 ng genomic DNA, 1 × PCR buffer (described above), 0.01 M Tris, 0.3 μM each primer (primer pairs covering the HHV-6 genome are shown in Sup. Table 1), 0.04 U/μl *Taq* polymerase (Kapa Biosystems) and 0.004 U/μl *Pwo* (Genaxxon Bioscience). Thermal cycling conditions were 96 °C for 2 minutes, followed by 30 or 32 cycles of 96 °C for 10 s, 56–62 °C for 30 s and 68 °C for 2–10 minutes (varied according to amplicon length), and then a final extension step at 68 °C for 10 minutes. Upon completion, 2 μl of each PCR was size-separated by agarose gel electrophoresis in the presence of 0.5 μg/ml ethidium bromide.

### Droplet-digital PCR

Droplet-digital PCR (ddPCR) copy number analysis was conducted on the QX200 platform (Bio-Rad Laboratories, Herts, UK). Duplex ddPCR assays incorporated a commercially available endogenous control assay for the human RPP30 gene (Bio-Rad Laboratories) and assays for the HHV-6 U38 (DNA polymerase) or HHV-6 U7 genes^40^. Copy number analysis of the HHV-6A DR6 gene (present in both DR_L_ and DR_R_) was conducted in the same manner using forward primer 5′-GAAACTGTAACGGCCACGTT-3′, reverse primer 5′-GTGCTCCGCCACGACTAC-3′ and probe 5′-HEX-CGCCGCCGCCGTTACTGTC-BHQ1-3′. In all three duplex assays, the reporter dyes were FAM and HEX for the control and viral components, respectively, with BHQ1 as a dark quencher. Prior to ddPCR, 1 μg of the DNA to be assayed for the U38 and DR6 genes was digested with 20 U *Hind*III. The RPP30/U7 reactions were performed using the previously described ‘in reaction’ digest technique^40^. The RPP30/U38 and RPP30/DR6 reactions were performed in duplicate, and the results were combined using the ‘merge’ function of the QuantaSoft analysis software v1.4.0. All reactions contained in excess of 10,000 droplets.

### Simple tandem repeat (STR) analysis

The primers used to amplify the D19S252, D19S246 and D19S254 STRs were described previously^55^. Each 10 μl PCR reaction consisted of 10 ng genomic DNA, 0.4 μM each primer, 1 × buffer A (Kapa Biosystems), 0.2 mM dNTPs and 0.05 U/μl *Taq* polymerase (Kapa Biosystems). Thermal cycling conditions were 96 °C for 2 minutes, followed by 28 cycles of 96 °C for 10 s, 56–62 °C for 30 s (the temperature depending on the STR), 68 °C for 2 minutes, and a final extension step of 68 °C for 10 minutes. PCR products were size-separated in a 3% MetaPhor (Lonza) agarose gel.

### Single telomere length analysis (STELA)

STELA was conducted as described previously, using genomic DNA (250 pg/reaction) for telomeres at 12q (12qSTELA primer), 17p (17p6 primer) and XpYp (XpYpE2 primer), and 500 pg/reaction for the ciHHV-6-associated telomere (DR1R primer)^25^^,^^34^,^56^. Individual amplicons, detected by phosphor-image analysis, were sized using the Imagequant software (Typhoon 9400, GE Healthcare) with known size markers (GeneRuler 1 kb and GeneRuler High Range DNA ladder, Fermentas), and the length of the flanking sequence was subtracted. The telomere lengths were presented as scatter plots with median and interquartile ranges. Telomere lengths in the blood and PEL samples were compared using the non-parametric Kruskal-Wallis test (GraphPad Prism; GraphPad Software Inc., CA, USA).

### Assays for iciHHV-6 truncations at DR_L_-T2 and extra-chromosomal circular HHV-6 DNA

Truncations in the T2 region of DR_L_ (DR_L_-T2) were detected by using STELA with the HHV-6A-specific UDL61R primer (Supplementary Table S1) and genomic DNA (1 ng/reaction). Control STELAs were performed at the same time with primer DR1R (0.5 ng/reaction). Extra-chromosomal circular HHV-6 DNA was detected essentially as described previously^25^, with minor modifications. Genomic DNA (90 ng/reaction) was amplified in 30 μl volumes by using primers UDL61R and U100Fw2 at 0.3 μM (primer positions shown in Fig. 1). Thermal cycling conditions were 96 °C for 1.5 minutes, followed by 26 cycles of 96 °C for 10 s, 62 °C for 30 s and 68 °C for 15 minutes, and then a final extension step of 68 °C for 10 minutes. Control PCRs using primers UDL61R/DR3F or U100Fw2/DR3R were conducted at the same time. Amplified products (20 μl) were size-separated in 0.8% agarose gels and detected by Southern blot hybridization to a DR3 probe.

## Additional Information

**Accession codes:** The sequence of the 1501-iciHHV-6A genome was deposited in GenBank under accession number KT355575.

**How to cite this article**: Zhang, E. *et al*. HHV-8-unrelated primary effusion-like lymphoma associated with clonal loss of inherited chromosomally-integrated human herpesvirus 6A from the telomere of chromosome 19q. *Sci. Rep.* **6**, 22730; doi: 10.1038/srep22730 (2016).

## Supplementary Material

Supplementary Information

## Figures and Tables

**Table 1:** Analysis of HHV-6A copy number using ddPCR.

Sample	U38 copies/cell^a^	U38 Poisson CI^b^ min-max	DR6 copies/cell^a^	DR6 Poisson CI^b^ min-max	Ratio DR6/U38	U7 copies/cell^a^	U7 Poisson CI^b^ min-max
1500-Bl	1.08^c^	1.03–1.13	2.16^c^	2.08–2.23	2.00	–	–
1500-T	0.11^c^	0.09–0.12	0.21^c^	0.20–0.22	1.99	0.09	0.08–0.10
1499	1.00	0.96–1.04	–	–	–	–	–
1501	1.03	0.99–1.07	–	–	–	–	–

^a^Copy number relative to the human RPP30 gene.

^b^CI, confidence interval.

^c^The U38 data, DR6 data and the CI were obtained from duplicate assays, and the data were merged as described in Materials and Methods.

**Table 2:** Comparison of telomere length in the patient’s blood (1500-Bl) and cells in the pleural fluid (1500-T).

Telomere	ciHHV-6 (DR1R)^a^		12q^a^		17p^a^		XpYp^a^	
Sample	Length	Range	Length	Range	Length	Range	Length	Range
1500-Bl	3158	2464–4220	4008	3102–5588	3560	2922–4410	5362	4119–6398
1500-T	3115	1766–4224	5987	2246–16103	6278	2949–16544	8337	3713–14826
p value^b^	0.8694^ns^	–	0.0002***	–	0.01**	–	<0.0001****	–

^a^Median telomere length and inter-quartile range (bp).

^b^The telomere length distributions in DNA from blood and cells in the pleural fluid were compared using a non-parametric Kolmogorov-Smirnov test; p values: ^ns^ not significant; *0.01 to 0.05 significant; **0.001 to 0.01 very significant; ***0.0001 to 0.001 and ****<0.0001 extremely significant.
