# Supplementary material for: HHV-8-unrelated primary effusion-like lymphoma associated with clonal loss of inherited chromosomally-integrated human herpesvirus-6A from the telomere of chromosome 19q
Source: Sci Rep. 2016 Mar 7;6:22730. doi: 10.1038/srep22730 (PMC4779988; doi:10.1038/srep22730)
Supplement: Supplementary Information [file srep22730-s1.pdf]

HHV-8-unrelated primary effusion-like lymphoma associated with clonal loss of inherited chromosomally-integrated human herpesvirus 6A from the telomere of chromosome 19q.

Enjie Zhang[1], Victoria E. Cotton[1], Alberto Hidalgo-Bravo[1], Yan Huang[1], Adam Bell[2], Ruth Jarrett[2], Gavin S. Wilkie[2], Andrew J. Davison[2], Ellie Nacheva[3], Reiner Sibert[4], Aneela Majid[5], Inga Kelpanides[5], Sandrine Jayne[5], Martin J.S. Dyer[5] and Nicola J. Royle[1*]

Supplementary Table S1 and Figures S1-S3

Supplementary Table S1 Primers

Primer name	Primer sequence	Primer position in reference U1102		Annealing temp.
DR1F HST/KUK	ACCTTGGCCCGAGCAAGAATGC	638	658	
DR8RA	GGATTACGGAGGTGAATGTTGC	7451	7430	62C
DR8F	GCAGAGACAAAAGTATGCGGAAG	7274	7296	
HHV6A probe 40R	GCGATTCTCGTATCGGGTTA	11913	11894	56C
HHV6A probe 51F	GGATACACCCCACTCCACAT	11666	11685	
HHV6A probe 51R	GAGTGCAATACAGAAGCCGG	17492	17473	60C
HHV6A probe 42 F	CCATTTCTTGCGAATGTTGA	17282	17301	
HHV6A probe 42 R	GATACGTCAAGACGGGGAAA	19068	19049	58C
HHV6 probe 5F1(U11)	TTTTTACATCACGACGCGATC	18958	18978	
HHV6 probe 5R	ATGGTCCTCCATGGGTTCTT	24962	24943	52C
HHV6 probe 18 F	ATGGCGCACGCTAAAAAG	22898	22914	
HHV6 probe 6R2	TGTAACGGAGGAATGGGAAG	30570	30551	56C
HHV6A probe 43 F	GGCAGTTGTCCAAAAATCTGA	30426	30446	
HHV6A probe 43 R	CCTGACTGTGGTCAACAACG	37177	37158	58C
HHV6 probe 17 F	TCGAACTAAACCCGAGACCT	37029	37048	
HHV6 probe 17 R	GCCATGTGGTTTGAGAGGAT	43487	43468	56C
HHV6 probe 7F	CGAAAAGCTCATGCTTACCC	43364	43383	
HHV6 probe 7R	AACTTGAAGCTGGCGACATT	49363	49344	56C
HHV6 probe 13 F	TCTCACTTCCGAAACTTCTATGC	49268	49290	
HHV6 probe 13 R	CAGTTGTTGCTTCCGATTCC	52664	52645	62C
HHV6A probe 52F	TATCCGCTCGAACACCAACT	52047	52066	
HHV6A probe 52R	CCAGCAAGAAAAGGAGCAGT	58242	58223	60C
HHV6 probe 15 F	CGTGACGTGTGCCAATCT	58062	58079	
HHV6 probe 15 R	GCTCAGTTGTCGAGGGAGAC	62168	62149	62C
HHV6A probe 50 F	CATCAAAAAGACAGCCAGGA	62039	62058	
HHV6A probe 50 R	GTTGGTATGGCCGAAGATCA	67338	67319	56C
HHV6 probe 32 F	ACACGCAACATGGCAAATAA	67168	67187	
HHV6 probe 32 R	CGTGCCATAGCGAAATGTAA	72346	72327	60C
HHV6A probe 49 F	CAGGAAAGGGACGGTGATAA	72178	72197	
HHV6A probe 49 R	ATCGAAAGCACCACCTTCAC	76670	76651	58C
HHV6A probe 30F2	GTGAAGGACCGTTGAGGTGT	76150	76169	
HHV6 probe 30 R	ATGCTTGCCTTTTCTCATGG	81675	81656	56C

Supplementary Table S1 (continued) Primers

Primer name	Primer sequence	Primer position in reference U1102		Annealing temp.
HHV6 probe 29 F	TGTCTCTCCTTCTGGCACCT	81594	81613	
HHV6 probe 29 R	GCAATCTTAGCAGCCGACTC	85341	85322	62C
HHV6A probe 47 F	GCTGTCGAGTCCACCATTTT	85168	85187	
HHV6A probe 47 R	TAACGTTCGGCGGAATTAAC	90530	90511	56C
HHV6 probe3 C1	CGCAGATAGCTTGTTGACCA	90465	90484	
HHV6 probe3 C2	CACTTCAGTTCCAGGGGTGT	96597	96578	56C
HHV6 probe 11 F	CGGAAACCATAGCTGTCCAT	96291	96310	
HHV6 probe 11 R	GCTTATGCTTCCCAATTCCA	100877	100858	52C
HHV6 probe 24 F	GCGGTAAACGGCATACATTT	100767	100786	
HHV6 probe 24 R	TGTACCTGGCAGCATCTGAG	103429	103410	58C
HHV6 probe 25 F	ATTGTTTATGCGTGCAGACG	103377	103396	
HHV6 probe 25 R	CCGTTGCTTTCTCTTCCATC	107612	107593	56C
HHV6 probe4 D1	GGGTTTAACGTAGCGAACCA	107521	107540	
HHV6 probe4 D2	CCGGAGAATGAAATCCTTGA	113415	113396	58C
HHV6A probe 41 F	TCGGACGTGTAAGATGTTGAA	113343	113363	
HHV6A probe 41 R	CGGATCACTCCCGAAATCTA	117406	117387	60C
HHV6A probe 44 F	GGTGGATTACGCCACTGTTT	117310	117329	
HHV6A probe 44 R	AAACTGCACGAAATCCGAAG	123286	123267	60C
HHV6 probe 9F (U83 A/BF)	GCGCAAACAATGTGCGTAGT	123087	123106	
HHV6 probe 9R	TCTCCTCTTCCGTTGACACC	128903	128884	56C
HHV6A probe 45 F	TATCTTTGGTCGGGGCTCTT	128732	128751	
HHV6A probe 45 R	TGATTGCAACAGTGATGGTACA	133181	133160	58C
HHV6 probe 21 F	CACATCTGTATGCTAATGATTGCT	133039	133062	
HHV6 probe 21 R	AGATTGATTGCACCCGAAAC	137011	136992	56C
HHV6 probe 27 F	CAAGGTGGAGGTTTCTTTGG	136954	136973	
HHV6 probe 27 R	AGGACCGTGTCCCATCATAG	141622	141603	60C
HHV6A probe 48 F	CTGGCCCAAAACAGAAATTG	141446	141465	
HHV6A probe 48 R	GAGAGTTTTCCATGGCCACA	145806	145787	58C
HHV6 probe 26 F	CATGGTGGTTCTCCTGTGTG	145628	145647	
HHV6 probe 26 R	GAGGGTGGGCACGTATTTTA	147897	147878	58C
HHV6A probe 46F	GGTCAGGTCCTCACGACAGT	147806	147825	
DR1R	GAAGAAGATGCGGTTGTCTTGTT	151965	151943	60C
UDL61R (HHV-6A specific)	TTTTCGCTCACGTGGCGGTCT	8534	8514	
U100Fw2	TATCTCCGAACATGATGCTG	149754	149773	
1501-Pac1R1	CCGGGGGATTTGACGTAATT	31/151264	12/151245	
U1102-150004F	ACTTAACATTTCGGAGGTAG	150003	150021	
12qSTELA	CGAAGCAGCATTCTCCTCAG			
17p6	GGCTGAACTATAGCCTCTGC			
XpYpE2	TTGTCTCAGGGTCCTAGTG			

Supplementary Figure S1. Cytogenetic analysis of PEL-like lymphoma (1500-T)

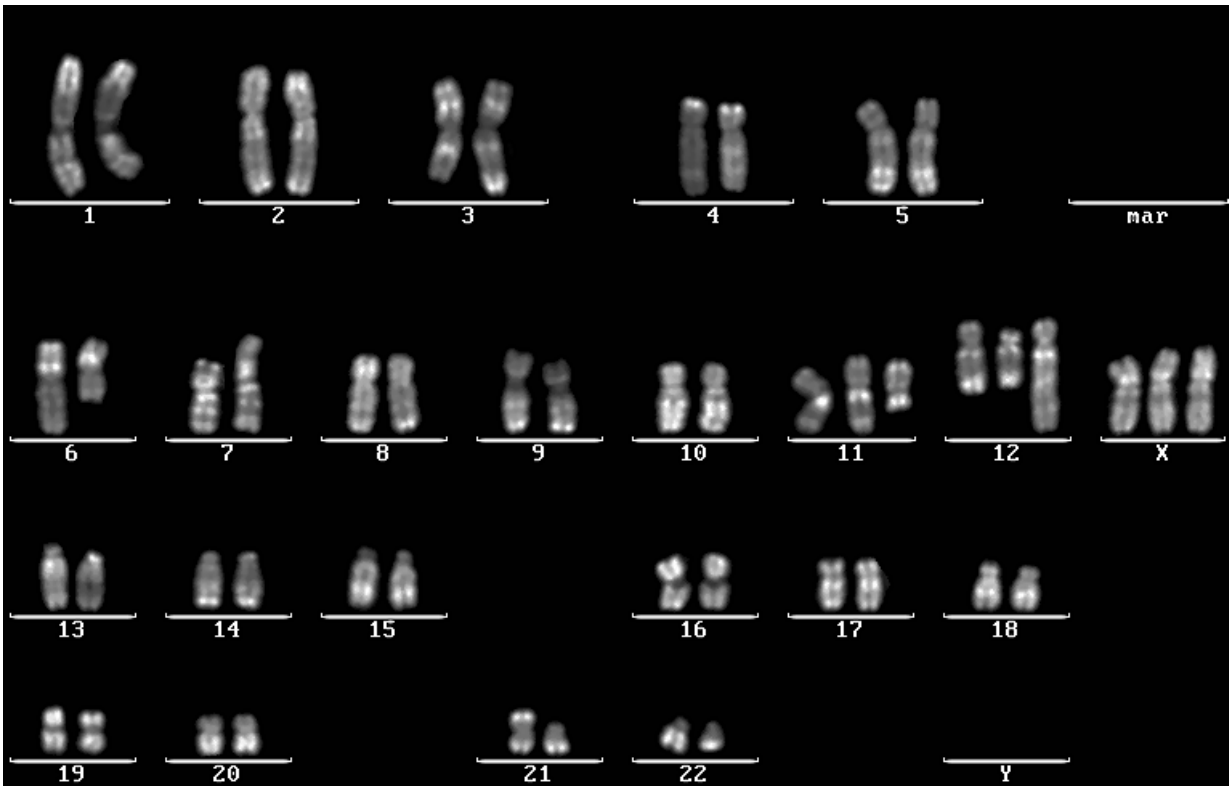

a) Karyotype: 48~49,XX,+X,der(2)t(2;12)(q14;q12), der(3)t(2;3)(q13;q11), t(3;22)(q27;q11),del(4)(q26q28),t(6;12)(p23;p12), del(6)(q14q22), add(7)(p14), +11,del(11)(q14), +12,der(12)t(4;12)(q13;q23~23)del(4)(q24q28),i(21)(q10)[cp30]

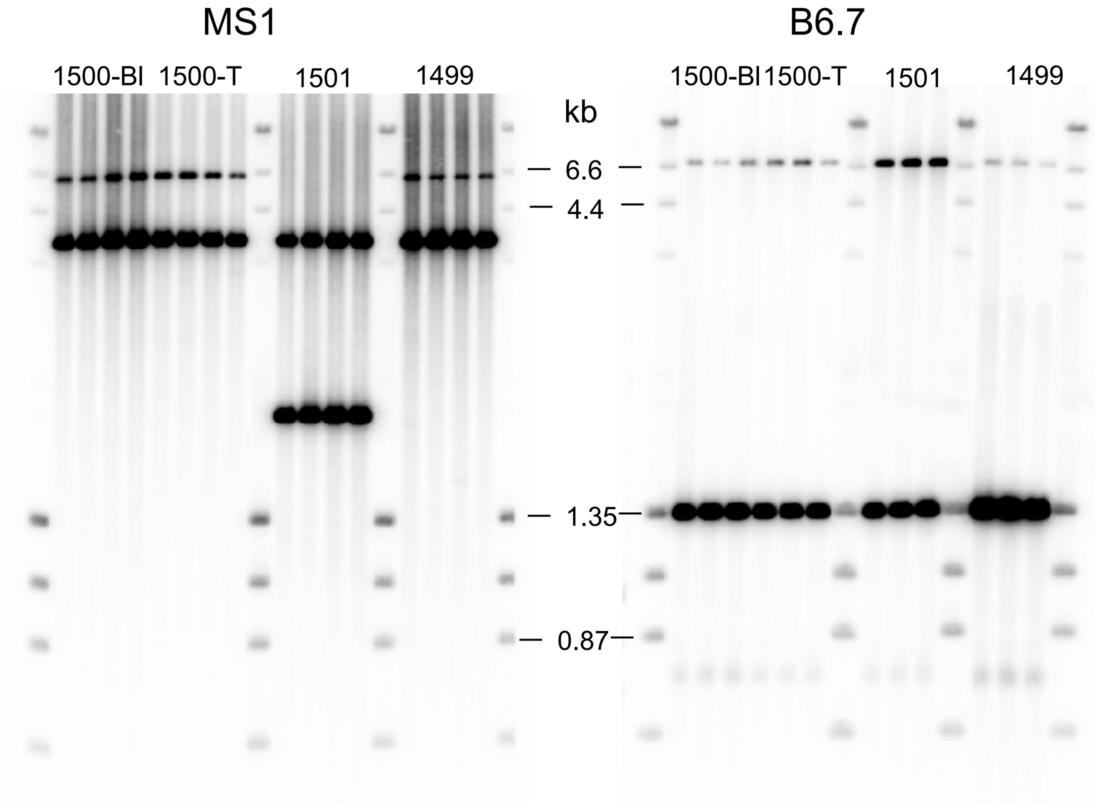

b) Genetic profiling of 1500 blood (1500-Bl) and PEL-like lymphoma (1500-T) samples

Supplementary Figure S2. Semi-quantitative analysis of HHV-6A amplicons in 1500 blood (Bl) and PEL-like lymphoma (T) DNA

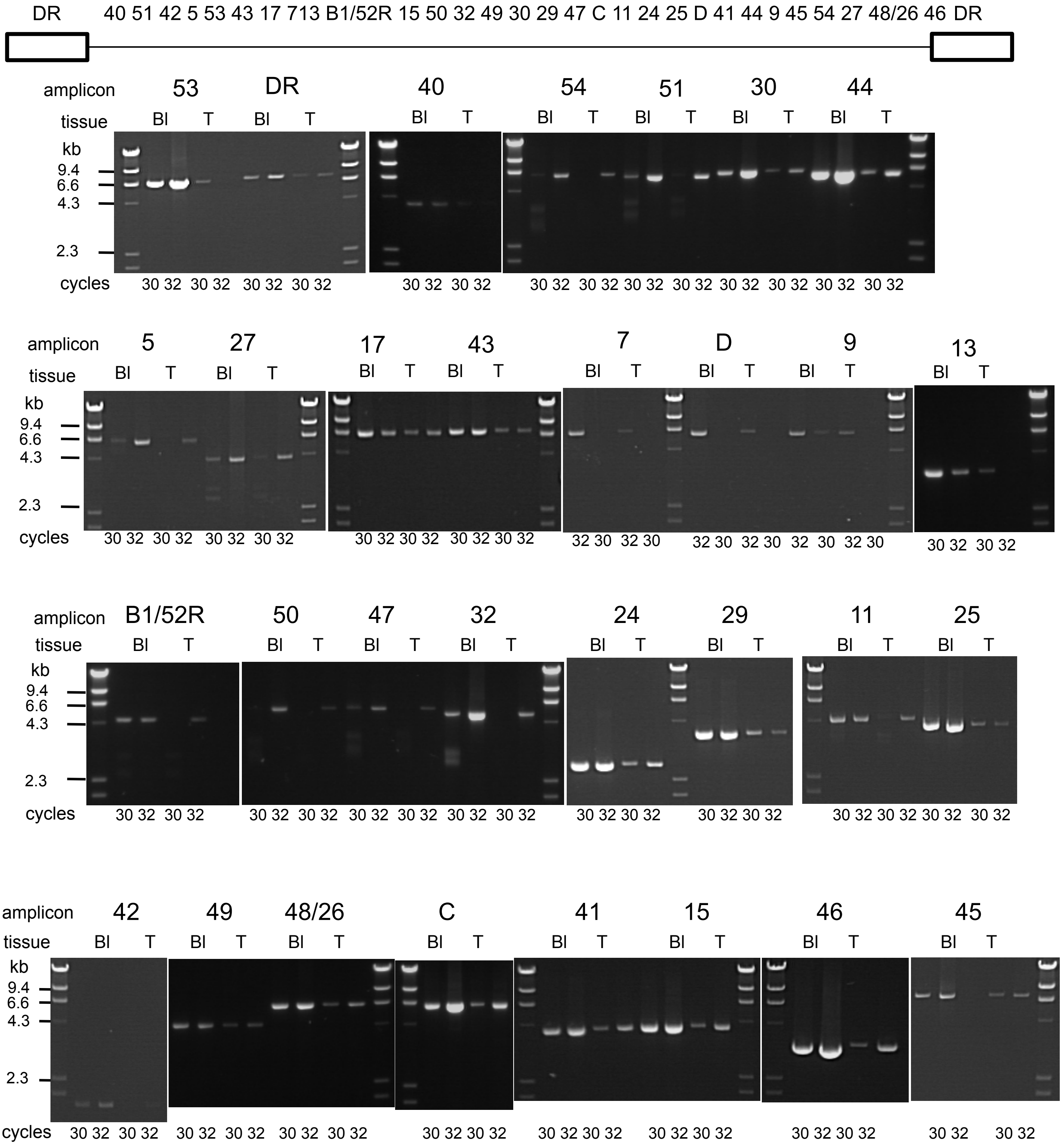

Supplementary Figure S3. Overlapping amplicons covering the iciHHV-6A genome

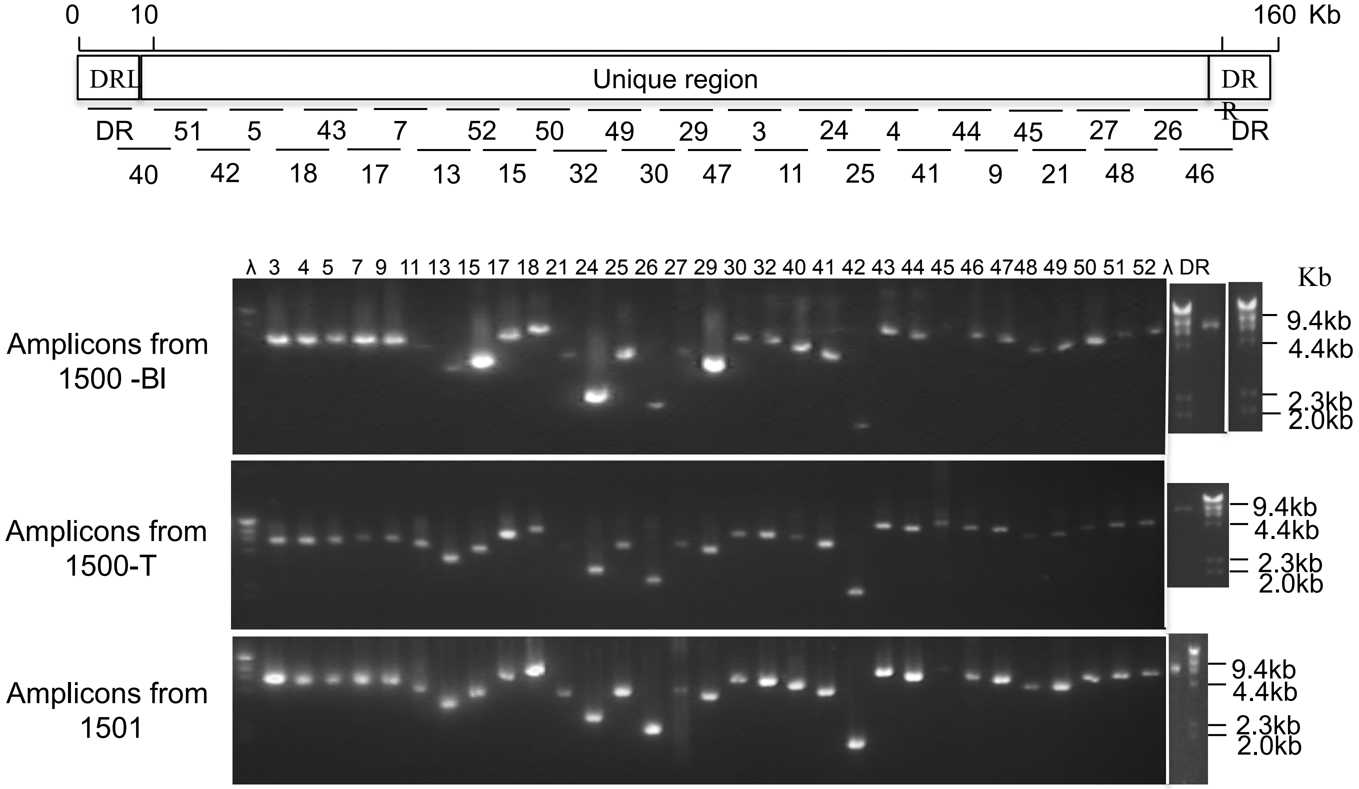

Overlapping amplicons covering the iciHHV-6A genome were generated from the patient's blood DNA (1500-Bl), the residual viral DNA in the PEL-lile lymphoma (1500-T) and from one brother (1501).
